# Hospital pharmacy response to the COVID-19 pandemic: experience from a regional referral center in the United Arab Emirates

**DOI:** 10.1186/s40545-023-00542-3

**Published:** 2023-03-02

**Authors:** Muhammad Hammad, Mohammad Mousa Tashtoush, El Mutasim Ahmed El Faki, Marwa Yousif Hajaj, Saima Saeed Ahmed, Amal Ahmed Darwish

**Affiliations:** Al Ain Hospital, Al Ain, United Arab Emirates

**Keywords:** COVID-19 pandemic, Hospital pharmacy during COVID-19 pandemic, Home medication delivery during COVID-19 pandemic, Clinical pharmacy during pandemic

## Abstract

**Background:**

The recent SARS‐CoV‐2 pandemic has resulted in significant morbidity and mortality worldwide. The healthcare systems, including pharmacies, faced unique challenges, such as managing  an overwhelming patient influx, clinical workforce management, transitioning to remote or online work, medication procurement and several others. The purpose of this study is to describe our hospital pharmacy’s experience dealing with the COVID-19 pandemic and to present solutions to the challenges that arose.

**Methodology:**

We retrospectively reviewed and consolidated strategies, interventions, and solutions that were implemented by our pharmaceutical institute in response to the challenges that arose during the COVID-19 pandemic. The study period was from March 1 to September 30, 2020.

**Results:**

We reviewed and organized our hospital pharmacy response to the COVID-19 pandemic into different categories. In inpatient and outpatient satisfaction surveys, physicians and patients expressed a high level of satisfaction with pharmacy services. The close collaboration between the pharmacy team and other clinicians was demonstrated through the number of pharmacist interventions, participation in the COVID-19 guidelines reviews, involvement in local and international research, and innovative solutions to inpatient and outpatient pharmacy medication management challenges.

**Conclusions:**

This study highlights the crucial role that our pharmacists and pharmaceutical institute played in ensuring continuity of care during the COVID-19 pandemic. We implemented several key initiatives, innovations, and collaborations with other clinical disciplines to successfully overcome the challenges faced.

## Introduction

In December 2019, a respiratory illness due to a novel coronavirus, SARS‐CoV‐2, was first identified in Wuhan, China [1]. Soon after that, on March 11, 2020, the World Health Organization (WHO) declared COVID-19 a pandemic [2]. At the time of writing this article, globally, there have been more than 642 million confirmed cases of COVID-19, including more than 6.6 million deaths, reported to WHO [3]. Over 1 million COVID-19 cases have been confirmed in the United Arab Emirates (UAE) to date, and there have been over 2300 deaths reported [4]. The disease severity of COVID-19 ranges from asymptomatic infection, mild illness to pneumonia, respiratory failure, critical illness and death [5–7]. During the time of this study, there was no specific treatment discovered against SARS‐CoV‐2. The management of COVID-19 patients largely involved symptomatic care, reducing the chances of complications, enhancing the immunity level of patients and the experimental use of repurposed drugs [7–11].

COVID-19 has had a negative influence on the regular provision of healthcare services worldwide [12, 13]. Emergency rooms, inpatients, and critical care units at the hospitals became overrun as more and more people caught the virus [13–15]. Healthcare systems across the world underwent at least some adaptations to manage the pandemic. This included creating temporary COVID-19 wards or dedicated facilities, increasing current capacity of general wards and intensive care units, etc. [13, 14, 16–18].

Due to the urgency of discovering treatment for sick patients and bringing the life back to normal, clinicians started using variety of traditional and non-traditional remedies. Practicing Evidence-Based Medicine (EBM) required enormous attention due to the fact that fresh information was always emerging. Carley et al. remarked, “The COVID-19 pandemic has arguably been one of the greatest challenges to EBM, since the term was coined in the last century” [19]. As the evidence for effective treatment against COVID-19 was lacking, misinformation and anecdotes were not only extensively disseminated through news, mainstream media and social media, but also practiced widely [19, 20]. A large number of clinical trials were started and preliminary or final results were being published gradually. There was an immense need to analyse the medical literature under the supervision of a multidisciplinary team of subject specialists for decision making. When the scientific evidence for treating COVID-19 began to emerge, clinical pharmacists played a crucial role in developing recommendations and implementing best practices [17, 20–23]. This role of clinical pharmacists became more important when newer molecules were introduced into the market as ‘investigational agents’ and for ‘emergency use authorization’ [22, 24].

Al Ain Hospital (AAH) originally had a capacity of 350 inpatient beds, including 14 adult intensive care unit (ICU) beds. Similar to other parts of the world, our healthcare system faced significant challenges during the pandemic. However, we persevered and adapted to the circumstances to ensure that our patients received the best care possible under difficult circumstances. To prevent the spread of the virus, several restrictions were imposed on daily activities along with additional, more stringent infection control precautions [4]. With increasing demands on isolation wards, inpatient wards, and ICU beds, the healthcare systems presented unique challenges [21, 25, 26]. In response to the pandemic crisis, our hospital’s emergency preparedness plan called for converting all inpatient beds to negative pressure rooms and increasing the ICU bed capacity to about 80 beds.

The pharmaceutical institute, AAH provides round-the-clock services. Clinical pharmacy, inpatient pharmacy, and outpatient pharmacy are the department’s three main sections. In addition, the department has sections for pharmacy informatics and inventory management. Our mission is to provide comprehensive and highest quality pharmaceutical services based on the best evidence to optimize drug therapy outcomes for our patients. Providing ongoing support to clinicians, particularly those who were redeployed to join the COVID-19 management teams, was critical for the pharmacy team. By doing so, our team played a vital role in ensuring that patients received the best possible pharmacotherapy throughout the pandemic. Moreover, our critical care bed capacity was increased fivefold. This necessitated crash courses for the newly joined physicians to quickly become familiar and work efficiently [27]. Clinical pharmacy team was resourceful to provide support by preparing quick drug summary sheets, dosage adjustments for renal or hepatic failure, guidelines etc. to facilitate safe medication prescribing and administration in ICU [28, 29]. Inpatient and outpatient pharmacies devoted their full attention to meeting the challenge of the COVID-19 pandemic. Complete restructuring of the services took place in a short amount of time and included extended hours of services, the incorporation of telemedicine patient counseling, modified discharge patient medication handling, pre-packaging of the protocol-driven medication bags, etc.

The pandemic presented a unique opportunity to solve new operational and clinical problems. It was a period of learning as well, since many of the challenges posed were outside of our routine work. In this article, we described how the pharmaceutical institute at a regional COVID-19 referral center dealt with different challenges related to medication therapy during the COVID-19 pandemic.

## Objective

The aim of this study is to retrospectively review, document, and emphasize the strategies and interventions implemented by the pharmaceutical institute in response to the COVID-19 pandemic at a regional referral centre.

## Methodology

This was a retrospective, descriptive study reviewing the documented interventions and strategies implemented by the pharmaceutical institute, Al Ain Hospital (AAH), during the COVID-19 pandemic. This included initiatives that were taken by the clinical pharmacy, inpatient pharmacy, outpatient pharmacy, pharmacy informatics and inventory management sections. The study period was from March 1 to September 30, 2020.

The pharmacy department adopted a multipronged approach to meet the challenges of the COVID-19 pandemic. This included but was not limited to revamping of clinical pharmacy, inpatient pharmacy, outpatient pharmacy, pharmacy informatics and inventory management. Details of the some of the innovations and responses are provided in the results sections.

We used following sources to collect data;

- Pharmacist Interventions documented in the electronic healthcare record system of the hospital
- Inpatient Satisfaction Survey (for physicians)
- Outpatient Satisfaction Survey (for patients)
- Internally documented achievements of inpatient pharmacy, outpatient pharmacy, clinical pharmacy, inventory management, pharmacy informatics and pharmacy administration during the COVID-19 pandemic.

The pharmacists’ clinical interventions that were documented in the electronic healthcare record system of the hospital, were extracted via system reports. Microsoft Excel spreadsheets were used  to store, sort and analyze data according to the types of intervention, clinical importance, and prescriber response. We performed satisfaction surveys for both inpatients and outpatients to determine the effectiveness of the initiatives taken during the pandemic.

## Results

We reviewed and organized our hospital pharmacy response to the COVID-19 pandemic into the following categories.

**Fig. 1:**
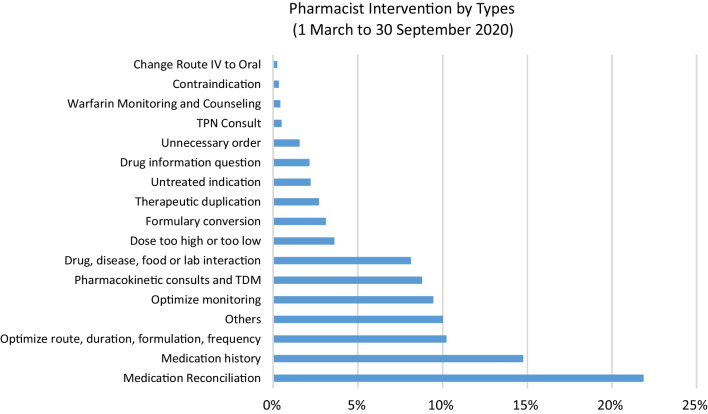
Pharmacist intervention by types (1 March to 30 September 2020)

**Fig. 2:**
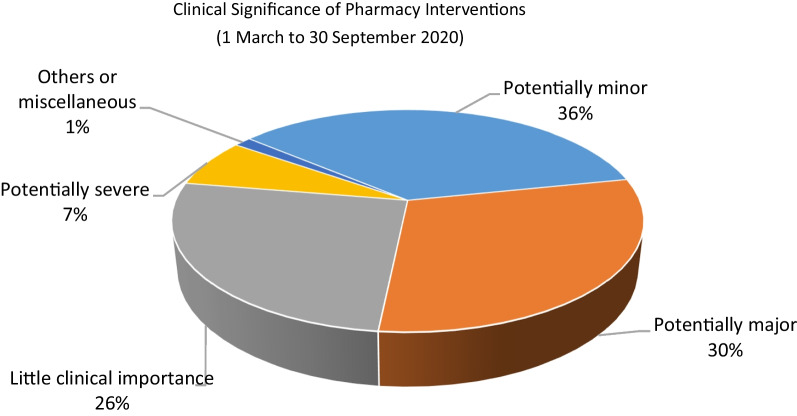
Clinical significance of pharmacist interventions (from 1 March to 30 September 2020)

1. Restructuring of clinical pharmacy team and distribution of duties
2. Analysis of the pharmacist clinical interventions related to COVID-19 patients
3. Contribution of clinical pharmacy team to the development of guidelines and protocol related to COVID-19 management and compliance audits
4. Inpatient pharmacy initiatives
5. Outpatient pharmacy initiatives
6. Satisfaction surveys
  - Inpatient (physicians)
  - Outpatient (patients)
7. Participation of the pharmaceutical institute in the COVID-19 clinical trials
8. Pharmacy informatics initiatives
9. Inventory management initiatives

1. Restructuring of the clinical pharmacy team and distribution of duties
  Clinical pharmacists were assigned to work with medical teams in COVID-19 wards and provide around-the-clock coverage. To mitigate the effects of staff shortage, we expeditiously provided training to three pharmacists from outpatient and inpatient pharmacies, enabling them to become members of our clinical pharmacy team. These pharmacists were promptly familiarized with the protocols and procedures related to the management of COVID-19 patients. To ensure comprehensive therapeutics-related support for medical, nursing, and allied healthcare teams and patients, the timings of clinical pharmacy services were extended from regular 8-h shifts (8 am to 4 pm) to a round-the-clock schedule, including weekends and public holidays. This decision was made due to the increased utilization of investigational or emergency use authorized medications, which posed a challenge for clinicians with limited experience in their utilization. The availability of the clinical pharmacy team on a 24/7 basis enabled the thorough review of patient profiles, identification of drug therapy-related issues, monitoring of parameters, and adherence to the most recent COVID-19 management guidelines and protocols through audits.
2. Analysis of pharmacist clinical interventions related to COVID-19 patients
  During the study period, a total of 11,893 main categories and 16,272 subcategories of pharmacy clinical interventions were documented. Figures. 1, 2 and 3 present a graphical representation of the interventions carried out by the pharmacists during this period. The most frequently documented interventions were medication reconciliation (21.87%), followed by medication history (14.76%) and optimizing route, duration, formulation or frequency (10.22%).

**Fig. 3:**
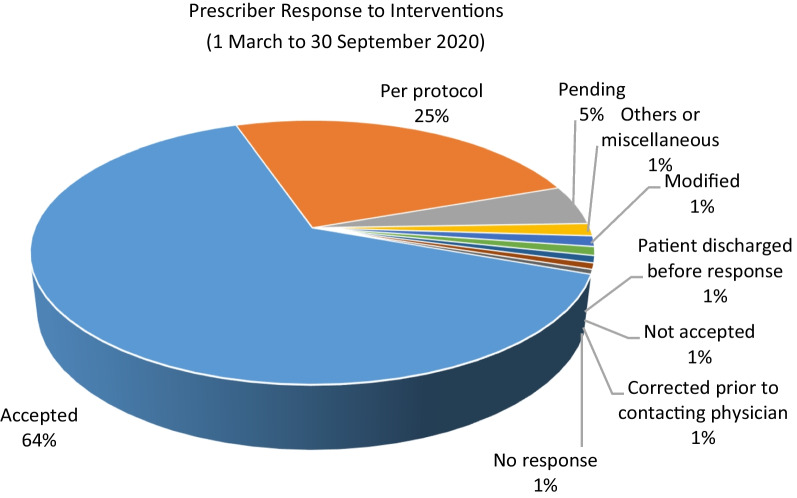
Prescriber response to interventions (1 March to 30 September 2020)
  In terms of clinical significance, the majority of interventions were categorized as potentially minor significance (36%), followed by potentially major (30%) and little importance (26%). It’s important to highlight that the majority of interventions (64%) were accepted by the prescribers, with an additional 25% responded to as per protocol. The pharmacist's recommendations were only not accepted by the physicians 1% of the time, which underscores the success of our collaborative approach to patient care.
3. Contribution of the clinical pharmacy team to COVID-19 management
  The clinical pharmacy team played a crucial role in ensuring adherence to the protocol-driven management and monitoring of COVID-19 patients. The following are some of the key areas in which the team made significant contributions:
  Literature review and guidelines development: as there was no FDA-approved drug or previous experience with the treatment of this viral disease, it was essential to keep abreast of the latest evidence-based management of COVID-19 and apply it consistently in practice wherever applicable. A committee was established to create and regularly update local hospital guidelines regarding diagnosis, patient flow, and management. These documents were frequently updated as new evidence emerged and aligned with the United Arab Emirates national guidelines. Clinical pharmacists worked closely with the COVID-19 hospital leadership and physicians to develop and update these guidelines. During the study period, the clinical pharmacy team, in collaboration with the hospital COVID-19 clinical guide team, produced a total of 14 versions of the COVID-19 treatment guidelines
  Reviewing the proper use of hydroxychloroquine (HCQ): given that HCQ has numerous drug interactions and is known to increase the QT interval on an electrocardiograph (ECG), the clinical pharmacy team, in collaboration with physicians, evaluated patients for contraindications to HCQ, potential drug interactions, electrolyte imbalances, and other factors to avoid adverse drug reactions. Patient education materials were also prepared to provide education on important aspects of safe medication use.
  Favipiravir: this was a new drug, not previously approved for use in the United Arab Emirates. It was known to cause teratogenicity, hepatotoxicity, increased uric acid levels and numerous drug interactions. The clinical pharmacy team assisted physicians by identifying contraindications, monitoring laboratory parameters, and educating patients on post-treatment monitoring and precautions. In addition, a patient education material was prepared for Favipiravir to provide important information about the use of this drug.
  Shift handovers: the clinical pharmacy service at our hospital transitioned to round-the-clock operations, and as a result, a handover process was implemented to ensure continuity of care by briefing the next shift teams with important patient-related updates.
  Venous Thromboembolism (VTE) Prophylaxis: the issue of VTE prophylaxis during the COVID-19 pandemic was a topic of ongoing debates in the literature, whether to use normal or increased doses of anticoagulants. Our clinical pharmacy team made a vital contribution by proactively identifying emerging evidence and implementing evidence-based practices guided by the latest guidelines. In addition, our hospital was a study site for a large multi-centre trial on the use of anticoagulants in COVID-19 patients [30].
  Antimicrobial Stewardship Program: our team, in collaboration with infectious disease physicians, was instrumental in reviewing antibiotic use and implementing an Antimicrobial Stewardship Program. In critical care units, a special module for renal dose adjustment was made available to clinicians as a quick reference, and empirical antibiotic guidelines pocket cards were distributed to standardize practices.
  Tocilizumab in severe COVID-19 patients: another therapeutic challenge in managing COVID-19 patients was identifying suitable candidates for treatment with tocilizumab. As the evidence was still developing, clinical pharmacists worked with medical and infectious disease teams to develop and update guidelines for prescribing tocilizumab.
  Dosage form optimization: our team also worked to optimize the dosage forms of nebulized medications by switching to handheld inhaler devices whenever possible and applicable, to avoid the spread of droplets.
  Electronic Healthcare Record (EHR) System: the team assisted in managing the electronic healthcare record system, updating medication order sentences, order sets, and patient education instructions.
  Appropriate use of Glucocorticoids: our team also reviewed patients with physicians to assess the appropriateness of using glucocorticoids and monitored for appropriate dosing, duration, blood glucose levels, electrolyte levels, and clinical improvement.
4. Inpatient pharmacy initiatives
  Our inpatient pharmacy operates round the clock. During the COVID-19 pandemic, inpatient pharmacy faced some unique challenges and adopted innovative solutions. Following are some of the important initiatives undertaken by inpatient pharmacy.
  Handling the shortage of personal protective equipment (PPE):
    Access to intravenous and sterile compounding clean room was minimized to only crucial staff (reduced to two shifts). Tasks such as checking, packaging and similar were shifted outside of the clean room. In addition, staff working the evening and night shifts only entered the clean room when the first preparation order was received.
  Managing the supply of medications as a unit dose:
    To minimize the risk of cross-contamination and the spread of infection, the use of medication trollies was suspended. Medications were provided to patients in disposable plastic bags, properly labelled with patient information. Upon receipt at the nursing unit, nurses sorted the medications in the existing cassette on the nursing floor, using at least two forms of patient identification. Any partially used or expired medications were promptly discarded in the nursing area. Unused medications were returned to the pharmacy and handled according to the process described below.
  Crash cart medication replenishment:
    To reduce the risk of exposure to COVID-19 and minimize unnecessary movement of personnel, crash cart medication trays were no longer transported to the pharmacy for replenishment. Instead, nurses would request the specific quantity of medications needed, and the pharmacy would provide them directly in the ward.
  Handling of patient’s own medications:
    As a general policy, our hospital discourages the use of patients’ own medications, with exceptions made for non-formulary or out-of-stock medications. To ensure the safe and appropriate use of patients’ own medications, the inpatient pharmacist assisted nurses in reviewing the medication for the generic name, brand name, strength, dosage form, integrity, and expiration date. After confirmation, the inpatient pharmacist verified the medication order as “patient’s own medication” in our electronic healthcare record system and sent the medication label to the ward.
  Recycling returned medication:
    The pandemic had a significant impact on the medication supply chain, leading to shortages and potential wastage of unused medications. To mitigate these issues, we proactively implemented measures to recycle returned and unused medications. A designated staff collected unused medications from all wards every Sunday. These medications were quarantined in a dedicated area of the pharmacy store. On Thursdays, pharmacy staff disinfected the items (that can be restocked) using 70% isopropyl alcohol under aseptic techniques in a negative pressure area using a vertical laminar airflow hood. The cleaned and sterilized medications were documented in a log sheet and recycled to prevent wastage and shortage.
  Addressing shortages of prefilled syringes:
    To address shortages of medications available as prefilled syringes (such as propofol, calcium chloride, etc.), the pharmacy prepared these syringes in-house as needed, rather than relying on external suppliers. This solution allowed us to adapt to the changing demand and ensure an adequate supply of these essential medications.
5. Outpatient pharmacy initiatives
  During the pandemic, all outpatient clinics transitioned to telemedicine, and the outpatient pharmacy department responded by offering a home delivery service for medication refills and prescriptions on a daily basis.
  In the morning, a list of patients due for refills was generated from the electronic healthcare record system. Assigned pharmacists verified medication orders and obtained any necessary insurance approvals. Narcotics, injectable medications, biologics, and drugs that require routine monitoring or patient assessment were excluded from the home delivery service. Cold chain maintenance was ensured during the transportation of medications. A text message (SMS) was sent to eligible patients 1 day before the distribution, notifying them of the upcoming home delivery. The home delivery company delivered medications to patients at their addresses after scanning their identification cards. Pharmacists provided tele-counselling to patients to ensure the medications were received and provide any necessary education.
6. Satisfaction survey:
  - Inpatient survey (physicians)
    A satisfaction survey of inpatient physicians consisting of 10 brief questions was conducted to evaluate the performance of the pharmacy department during the COVID-19 epidemic. A total of 22 physicians, including internal medicine consultants, specialists and general practitioners responded to the survey questions. The results of the survey are as following (Figs. 4, 5, 6, 7, 8, 9, 10, 11, 12, and 13):

**Fig. 4:**
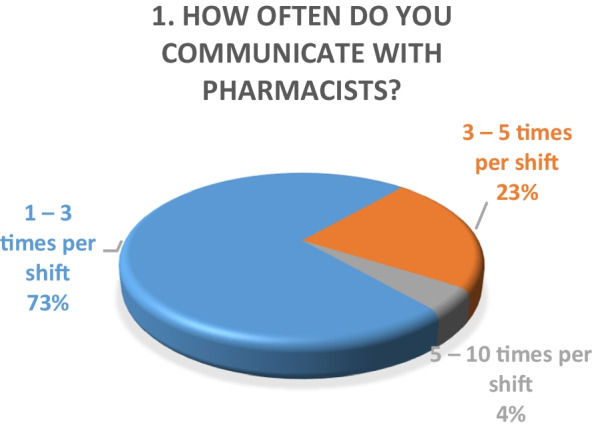
Result of survey question 1

**Fig. 5:**
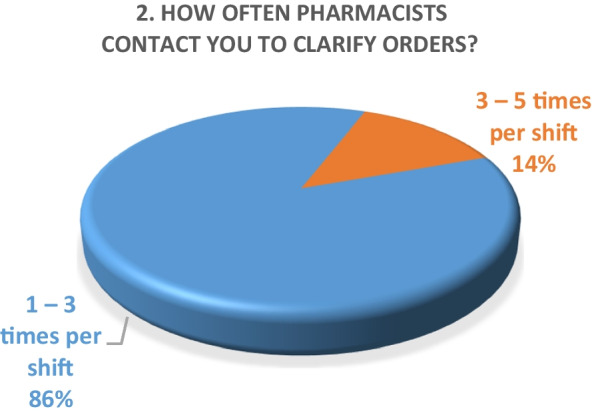
Result of survey question 2

**Fig. 6:**
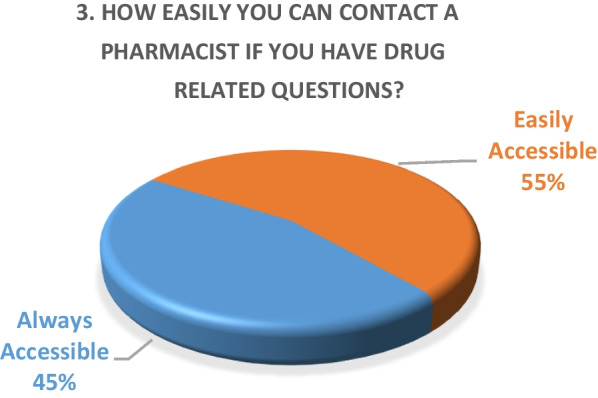
Result of survey question 3

**Fig. 7:**
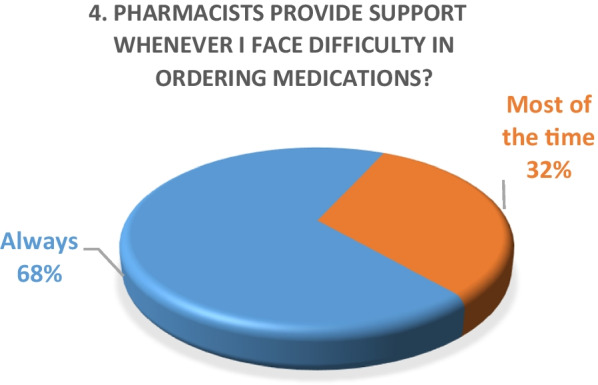
Result of survey question 4

**Fig. 8:**
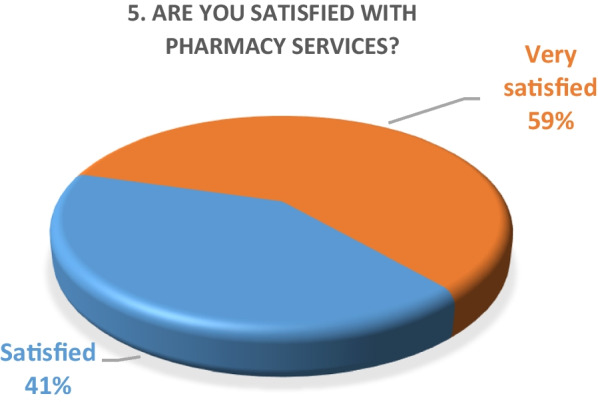
Result of survey question 5

**Fig. 9:**
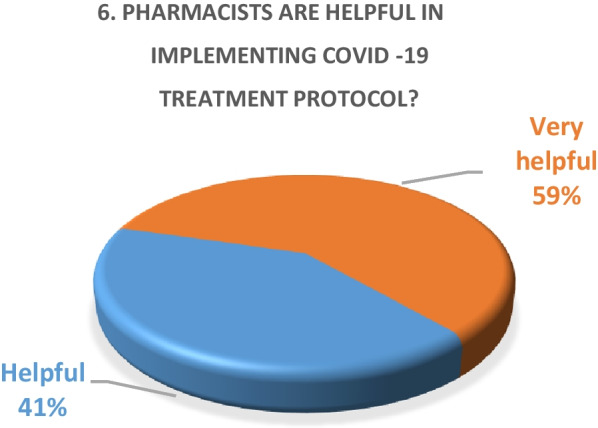
Result of survey question 6

**Fig. 10:**
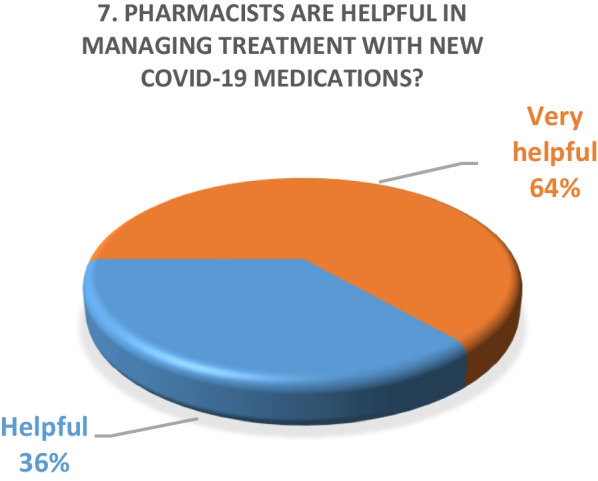
Result of survey question 7

**Fig. 11:**
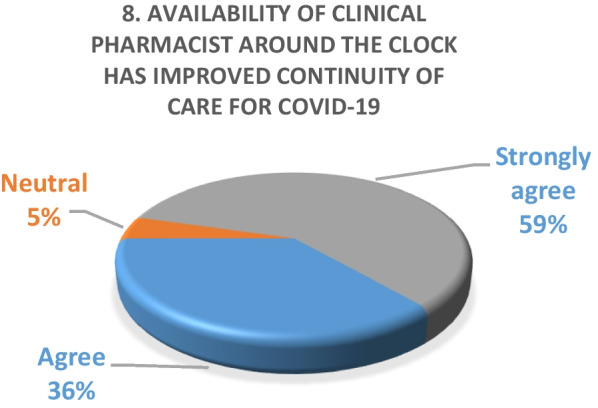
Result of survey question 8

**Fig. 12:**
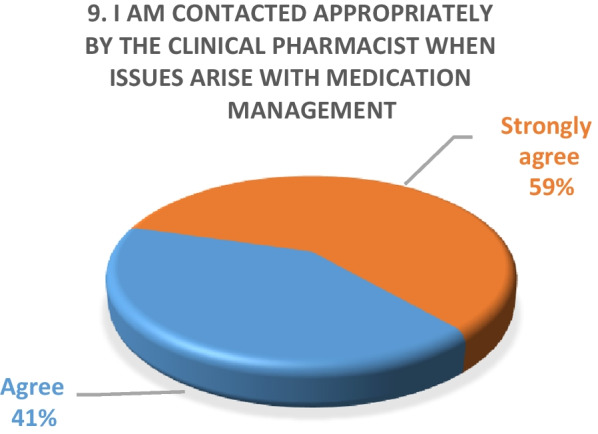
Result of survey question 9

**Fig. 13:**
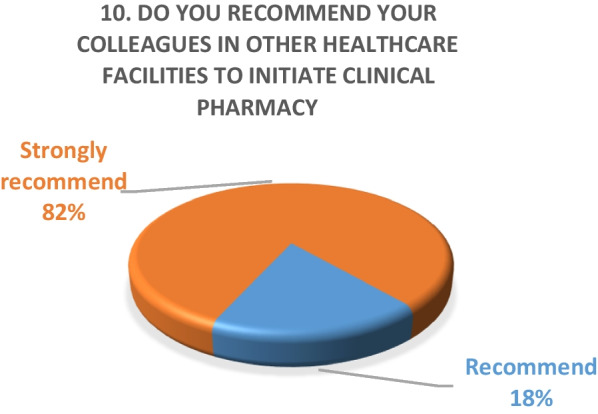
Result of survey question 10
    A majority of the physicians were “very satisfied” (59%) and the rest (49%) were “satisfied” with the performance of the pharmacy department.
  - Outpatient pharmacy survey
    From March 30th to June 30th 2020, a total of 44,242 medication orders were processed and delivered. In addition, over 4700 patients received tele-counseling for 21,244 medications during this time period (Fig. 14).

**Fig. 14:**
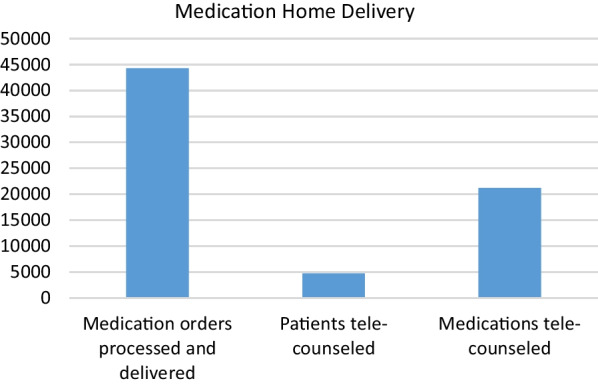
Tele-counseling
    To evaluate patient satisfaction with the medication home delivery service implemented during the pandemic, a survey was conducted with 1005 patients. A simple 10-question survey was administered to patients. The results of the survey indicated that overall, 68% (684) of patients were “very satisfied” and 30% (298) were “satisfied” with the service. Regarding pharmacist tele-counseling, 65% (652) were “very satisfied” and 32% (320) were “satisfied”. Furthermore, 75% (754) of patients were “very satisfied” with the delivery experience, and 25% (251) were “satisfied”. A majority of the surveyed patients, 95% (951), recommended that the delivery service should be continued.
7. Participation of the pharmacy department in the COVID-19 trials
  Our pharmacists were actively involved in several important national and international clinical research projects related to the management of COVID-19. Some of this research has already been published, while other studies are still ongoing. The clinical pharmacy team participated in the published randomized international multicentre trial [30] and epidemiological study [31].
8. Pharmacy informatics initiatives
  Our pharmacy informatics team was quick to implement changes to the electronic healthcare system as new evidence emerged regarding the repurposing of old medications and the emergency use authorization of new antivirals. Some of the major tasks undertaken by the team included building, modifying, and deleting product descriptions in the system, developing medication order sentences, updating drug interactions and safety alerts, developing and updating order sets, generating medication consumption reports, therapeutic drug monitoring, and intravenous to oral switch reports, among others.
  The informatics team created and regularly updated order sets for standard medication packages for discharged patients. This not only helped physicians to prescribe home medications quickly with fewer clicks, but also relieved the workload of inpatient and outpatient pharmacy by pre-emptively preparing standard bags for dispensation.
9. Procurement and inventory management initiatives:

To ensure the continuity of medication supply and minimize stock shortages, our procurement team implemented several strategies, such as:

- Collaborated with the corporate pharmacy team and other healthcare facilities to secure a steady supply of medications.
- Reviewed medication consumption forecasts in collaboration with clinical pharmacists and physicians to manage inventories accordingly.
- Identified alternative sources of medication in case of unavailability from regular suppliers.
- Regularly monitored the inventory levels of essential medications and quickly replenishing as needed.
- Implemented strict measures to ensure the integrity and safety of medications during transportation and storage.

These efforts helped us to minimize disruptions in medication supply and ensure that our patients had access to the medications they needed, especially during the challenging time of the pandemic.

## Discussion

The COVID-19 pandemic had a significant impact on the healthcare sector worldwide. To accommodate the surge of patients, many hospitals had to change their workflow. There have been few published studies that have addressed the role of pharmacists during this pandemic. A recent survey showed how hospital pharmacies in the United States implemented drastic changes related to medication management and use (MMU) system during the COVID-19 pandemic [32].

The COVID-19 pandemic presented many challenges for governments and healthcare systems worldwide. The United Arab Emirates was one of the countries that responded quickly and efficiently, deploying a large number of resources to combat the virus [33]. This included implementing strict quarantine measures, increasing testing, and supporting public health initiatives. These efforts helped the country to effectively manage the spread of the virus and keep the number of cases to remarkably low [33, 34]. The utilization of emerging therapeutic interventions highlighted the crucial role of pharmacy services, which encompassed a range of activities, including medication procurement, pharmacotherapy, and monitoring.

A semi-structured qualitative survey conducted among pharmacists working in clinical roles across Europe revealed that the routine functioning of clinical services was disrupted, and many tasks were performed remotely [35]. Our clinical pharmacy team similarly experienced this shift towards remote and online modes of provision. Earlier surfaced literature proposed some therapeutic areas, where clinical pharmacists can play key roles [36]. These included antiviral stewardship, safe and effective use of repurposed and investigational drugs for COVID-19 infection, patient counselling, mitigating drug shortages by proper consumption forecasting, identifying effective therapeutic alternatives in case of drug shortages, prioritizing drug supply to the patients who are most likely to benefit and formulary restriction criteria for COVID-19 therapies that are in limited supply. Our clinical pharmacy team actively contributed in all these key areas to facilitate the desired functioning of the hospital. Data of our pharmacist clinical interventions showed that the highest interventions were related to medication reconciliation (21.87%) followed by medication history (14.75%) and optimize route, duration, formulation or frequency (10.22%). This demonstrates the active involvement of the pharmacy team in the continuity of care, since a large number of the patients with various disease states were transferred to our hospital, when diagnosed with COVID-19. With respect to prescriber response to pharmacist interventions, 64% were accepted, while 25% were per protocol, while only 1% of the recommendations were not accepted, reflecting a very high acceptance rate.

The results of both our inpatient and outpatient satisfaction surveys indicate that the pharmacy department performed exceptionally well in response to the COVID-19 pandemic situation. Participants in the inpatient survey were physicians, who reported frequent communication with pharmacists and high levels of satisfaction with their contributions. Pharmacists were easily or always accessible. The other survey evaluated patient satisfaction with a newly launched medication home delivery service in outpatient pharmacies. A majority (75%) of patients were either very satisfied or satisfied with the delivery experience. This suggests that the new approach to medication handling in the outpatient pharmacy was beneficial for patients during the challenging pandemic situation.

To the best of our knowledge, this is the first report from our country documenting the personal experience of pharmacy practitioners managing the COVID-19 situation. We aimed to include several important aspects and challenges faced by pharmacists during the pandemic. This study has few limitations. First, it is based on the experience of a single hospital pharmacy. A larger sample of similar centres may provide a more comprehensive understanding of how crisis situations were managed during COVID-19 pandemic. In addition, the study was conducted before the introduction of COVID-19 vaccines and monoclonal antibodies into clinical practice. These preventative and therapeutic options have significantly impacted the treatment of COVID-19 and the role of pharmacists.

## Conclusion

We reviewed and compiled some of the innovative strategies developed by the pharmaceutical institute of our hospital to ensure continuity of services during the COVID-19 pandemic. The clinical pharmacy team played a crucial role in evaluating emerging evidence on COVID-19 treatment and contributed to the development of relevant guidelines in collaboration with other clinical disciplines. Both inpatient and outpatient pharmacies adapted a number of workflows and processes to ensure smooth functioning of the medication management system in the hospital. The dynamic support provided by the pharmacy informatics team facilitated the swift implementation of guidelines and evidence-based practices. The results of both inpatient and outpatient satisfaction surveys indicate that the pharmacy team not only met the challenges posed by the pandemic but excelled in several areas. Our experience may serve as a useful reference for pharmacists in the medication management of future pandemics.

## Data Availability

Not applicable.
